# Roles for common MLL/COMPASS subunits and the 19S proteasome in regulating CIITA pIV and MHC class II gene expression and promoter methylation

**DOI:** 10.1186/1756-8935-3-5

**Published:** 2010-02-04

**Authors:** Olivia I Koues, Ninad T Mehta, Agnieszka D Truax, R Kyle Dudley, Jeanne K Brooks, Susanna F Greer

**Affiliations:** 1 Division of Molecular Genetics and Biochemistry, Georgia State University, Atlanta, Georgia, USA; 2 Division of Cellular and Molecular Biology and Physiology, Georgia State University, Atlanta, Georgia, USA; 3 Division of Biotechnology, Department of Biology, Georgia State University, Atlanta, Georgia, USA

## Abstract

**Background:**

Studies indicate that the 19S proteasome contributes to chromatin reorganization, independent of the role the proteasome plays in protein degradation. We have previously shown that components of the 19S proteasome are crucial for regulating inducible histone activation events in mammalian cells. The 19S ATPase Sug1 binds to histone-remodeling enzymes, and in the absence of Sug1, a subset of activating epigenetic modifications including histone H3 acetylation, H3 lysine 4 trimethylation and H3 arginine 17 dimethylation are inhibited at cytokine-inducible major histocompatibilty complex (MHC)-II and class II transactivator (CIITA) promoters, implicating Sug1 in events required to initiate mammalian transcription.

**Results:**

Our previous studies indicate that H3 lysine 4 trimethylation at cytokine-inducible MHC-II and CIITA promoters is dependent on proteolytic-independent functions of 19S ATPases. In this report, we show that multiple common subunits of the mixed lineage leukemia (MLL)/complex of proteins associated with Set I (COMPASS) complexes bind to the inducible MHC-II and CIITA promoters; that overexpressing a single common MLL/COMPASS subunit significantly enhances promoter activity and MHC-II *HLA-DRA *expression; and that these common subunits are important for H3 lysine 4 trimethylation at MHC-II and CIITA promoters. In addition, we show that H3 lysine 27 trimethylation, which is inversely correlated with H3 lysine 4 trimethylation, is significantly elevated in the presence of diminished 19S ATPase Sug1.

**Conclusion:**

Taken together, these experiments suggest that the 19S proteasome plays a crucial role in the initial reorganization of events enabling the relaxation of the repressive chromatin structure surrounding inducible promoters.

## Background

Di- or trimethylation (hypermethylation) of histone H3 at lysine 4 (H3K4) is found at actively transcribed genes [1-5], is functionally linked to activating acetylation events at promoters [6], and is mediated by the mixed lineage leukemia (MLL)/complex of proteins associated with Set I (COMPASS)-like complexes [7,8]. MLL/COMPASS-like complexes contain several well-characterized, common subunits; Ash2L, WDR5 and RbBP5, which are conserved between yeast and humans [9-11]. These subunits are thought to constitute a common MLL/COMPASS subcomplex that forms a platform to mediate the Set1 enzyme and H3K4 substrate interaction [9,11-13]. H3K4 hypermethylation also has been found to inhibit H3K9 methylation, and more recently has been associated with a release from repressive H3K27 methylation by MLL/COMPASS-associated histone demethylases [14-19]. Observations that the deposition of activating methylation events on H3K4 correlates with the removal of silencing methylation events on H3K27 have important implications for the regulation of bivalent genes, including cytokine-inducible genes of the immune system.

Major histocompatibility class (MHC)-II molecules are cell surface glycoproteins that present antigenic peptides to CD4^+ ^T lymphocytes of the adaptive immune system [20,21]. MHC-II expression is regulated primarily at the level of transcription by several well-studied elements within the MHC-II proximal promoter, which bind a series of ubiquitously expressed transcription factors to form a basal enhanceosome complex (see Additional file 1) [22-24]. Once assembled, the enhanceosome recruits a master regulator, the class II transactivator (CIITA) [25]. Transcription of CIITA is driven by one of four distinct promoters in a cell-type and cytokine specific manner. The well-characterized CIITA promoter (p)IV is responsible for interferon (IFN)-γ-inducible expression in nucleated cells [26,27]. Upon cytokine stimulation, the Janus kinase/signal transducer and activator of transcription 1 (JAK/STAT) pathway is triggered, leading to pIV accumulation of the requisite transcription factors, and the initiation of CIITA transcription [28-30]. Once recruited to the MHC-II promoter, CIITA binding stabilizes the enhanceosome complex and recruits basal transcriptional components to initiate the switch to an elongation complex [24,31-34]. Although much is known about the requirement of transcription factors at these cytokine-inducible promoters, considerably less is understood about the coordination of the histone modifying events that allow these promoters to switch from a semi-poised state to a fully open, transcription-accessible structure.

The objective of this study was to investigate the importance of 19S ATPase-regulated activating histone H3 trimethylation at lysine 4 (H3K4me3) events in chromatin remodeling at MHC-II and CIITA promoters, and to determine if the 19S ATPase Sug1directly affects multiple promoter remodeling events. In this report, we show that common MLL/COMPASS subunits (Ash2L, RbBP5 and WDR5) bind to the MHC-II promoter and to CIITA pIV; that overexpressing a single common subunit significantly enhances promoter activity and MHC-II *HLA-DRA *expression; and that WDR5 knockdown decreases H3K4me3 at promoters of both MHC-II and CIITA. Although we have previously shown H3K18 acetylation to be regulated by 19S ATPases, there were no observable changes in levels of H3K18 acetylation in the absence of WDR5, indicating that Sug1 independently regulates acetylation and methylation events. Further supporting our hypothesis that Sug1 regulates multiple independent events is our observation that knockdown of Sug1 promotes elevated H3K27me3 levels at cytokine-inducible promoters. Together these results implicate the 19S proteasome in the initial reorganization of chromatin to relax the repressive histone environment surrounding cytokine-inducible promoters.

## Results

### Common MLL/COMPASS subunits associate with cytokine-inducible promoters

**Figure 1:**
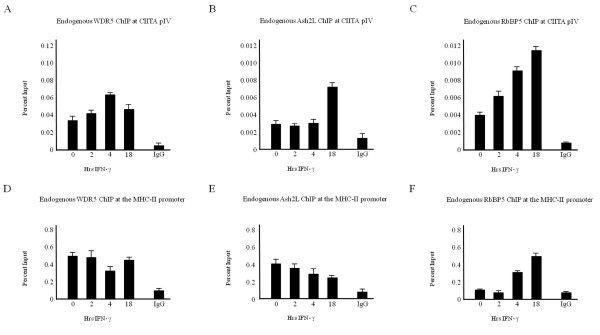
**Common MLL/COMPASS subunits associate with cytokine-inducible genes**. (a-c) Several common subunits of MLL/COMPASS associate with CIITA pIV. ChIP assays were carried out in HeLa cells stimulated with IFN-γ for 0 to 18 hours. Lysates were immunoprecipitated with control or endogenous (a) WDR5, (b) Ash2L or (c) RbBP5 antibody. Associated DNA was isolated and analyzed via real-time PCR using primers and probe spanning CIITA pIV. (d-f) Subunits of MLL/COMPASS associate with the MHC-II proximal promoter. ChIP assays were carried out in HeLa cells stimulated with IFN-γ for 0 to 18 hours. Lysates were immunoprecipitated with control or endogenous (d) WDR5, (e) Ash2L or (f) RbBP5 antibody. Associated DNA was isolated and analyzed via real-time PCR using primers and probe spanning the MHC-II proximal promoter. To demonstrate constitutive binding, data are presented as percentage input. IgG isotype control values were 0.001 ± 0.0005 (CIITA pIV) and 0.01 ± 0.007 (MHC-II promoter). Values represent mean ± SEM of (n = 3) independent experiments.

H3K4me3 is mediated by histone methyltransferase (HMTase) enzymes, which are typically recruited to DNA as part of a larger complex of proteins MLL/COMPASS [7,8,10,35]. We previously showed that the 19S ATPase Sug1 positively regulates H3K4me3 at the MHC-II proximal promoter and at CIITA pIV, potentially by stabilizing the association of common MLL/COMPASS subunits [36]. We observed that the common MLL/COMPASS subunit WDR5 binds to CIITA pIV with a moderate enhancement in recruitment observed upon cytokine stimulation, which is lost upon Sug1 knockdown [36]. To expand on the potential for MLL/COMPASS binding to CIITA pIV, we performed chromatin immunoprecipitation (ChIP) assays to determine if additional common MLL/COMPASS subunits also bind to CIITA pIV (Figure 1a-c). WDR5 (Figure 1a), Ash2L (Figure 1b) and RbBP5 (Figure 1c) associate with CIITA pIV in unstimulated cells, and levels of association are moderately enhanced upon prolonged cytokine stimulation. To determine if these common MLL/COMPASS subunits are recruited to the MHC-II proximal promoter, similar ChIP assays were performed (Figure 1d-f). Each of the three subunits, WDR5 (Figure 1d), Ash2L (Figure 1e) and RbBP5 (Figure 1f), bound to the MHC-II promoter in both unstimulated and stimulated cells, suggesting that these common subunits of MLL/COMPASS complexes form a backbone complex to allow H3K4me3 at these cytokine-inducible genes. It is noteworthy that these common MLL/COMPASS subunits are significantly associated with these cytokine-inducible promoters in unstimulated cells, as elevated levels of H3K4me3 have been reported previously [36-40] to accumulate only upon prolonged stimulation with IFN γ. All three of these subunits also bound to the constitutively expressed GAPDH promoter (see Additional file 2).

### Overexpressing a single common subunit of MLL/COMPASS complexes enhances CIITA transactivity and MHC-II *HLA-DRA *expression

**Figure 2:**
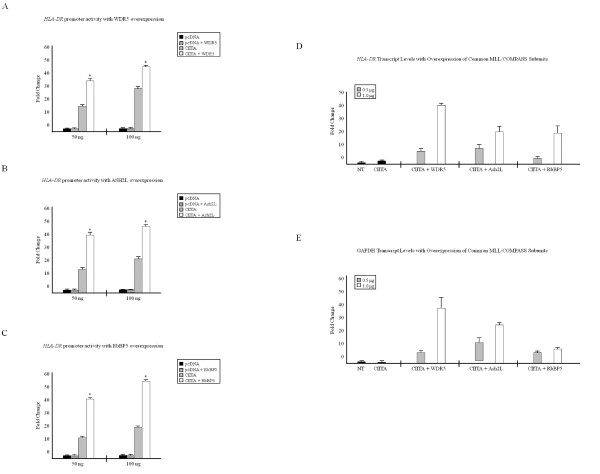
**Overexpressing a single common subunit of MLL/COMPASS complexes enhances CIITA transactivity**. (A-C) Overexpression of common MLL/COMPASS subunits increases CIITA transactivity at the MHC-II promoter. HeLa cells were transfected with *HLA-DRA*-Luc, *Renilla*, pcDNA3, CIITA and (a) WDR5, (b) Ash2L or (c) RbBP5. Luciferase activity is reported as fold activation relative to that of the reporter alone. Luciferase readings were normalized to *Renilla *activity. Assays were performed in triplicate and values represent mean ± SEM of (n = 3) independent experiments. (d-e) Overexpression of common MLL/COMPASS subunits increases MHC-II transcript levels. HeLa cells were either left untransfected or were transfected with CIITA alone or in combination with WDR5, Ash2L or RbBP5. RNA was prepared using TRIzol reagent and cDNA was generated using gene specific antisense primers for (d) MHC-II *HLA-DR *and **(e) **GAPDH. cDNA was quantified via real-time PCR, and data are graphed as fold changes over non-transfected samples. Values represent mean ± SEM of (n = 2) independent experiments. *P < 0.05 vs control siRNA.

To characterize the effect these common subunits of MLL/COMPASS have on the MHC-II proximal promoter, we performed luciferase reporter assays in unstimulated HeLa cells, in which the MHC-II *HLA-DRA *promoter is fused to a luciferase gene and is expressed along with CIITA and common MLL/COMPASS subunits WDR5 (Figure 2a), Ash2L (Figure 2b) or RbBP5 (Figure 2c). In cells transfected with CIITA alone, there was a 15-20-fold increase in *HLA-DRA *promoter activity (Figure 2a-c, gray bars), whereas overexpressing a single common MLL/COMPASS subunit (Figure 2a-c, white bars) significantly enhanced the ability of CIITA to drive the *HLA-DRA *promoter. Similarly, overexpressing a single common MLL/COMPASS subunit dramatically enhanced MHC-II *HLA-DRA *(Figure 2d) and GAPDH transcript levels (Figure 2e).

### A WDR5-dependent complex trimethylates H3K4 at cytokine-inducible genes

**Figure 3:**
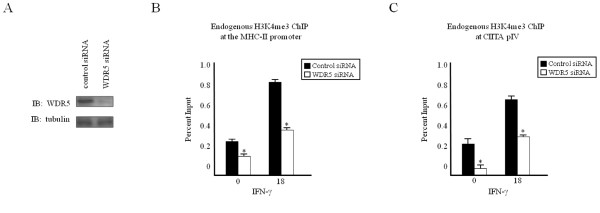
**Knockdown of a common MLL/COMPASS subunit decreases H3K4me3**. (a) WDR5 knockdown efficiently reduces WDR5 expression. HeLa cells were transfected with control or WDR5-specific siRNA, harvested and subjected to western blot analysis for (top) endogenous WDR5 and (bottom) endogenous tubulin. (b-c) WDR5 knockdown decreases H3K4me3. HeLa cells transfected with scrambled control or WDR5-specific siRNA were stimulated with IFN-γ and subjected to ChIP assay. Lysates were immunoprecipitated with control or endogenous H3K4me3 antibody. Associated DNA was isolated and analyzed via real-time PCR as described in Figure 1, using primers and probes specific for (b) the MHC-II promoter or (c) CIITA pIV. Data are presented as percentage input. IgG Isotype control values were 0.06 ± 0.03 (MHC-II promoter) and 0.003 ± 0.002 (CIITA pIV). Values represent mean ± SEM of (n = 3) independent experiments. *P < 0.05 vs control siRNA.

We observed that various common MLL/COMPASS subunits are recruited to both the MHC-II proximal promoter and to CIITA pIV (Figure 1), and that overexpressing these subunits can drive CIITA-dependent MHC-II gene expression (Figure 2). As these subunits are thought to constitute a common MLL/COMPASS subcomplex [9,11-13], we employed RNA interference to determine the contribution of WDR5 to the trimethylation of histone H3 lysine 4 at these genes. ChIP assays were performed in HeLa cells transfected with small interfering (si)RNA specific for the common MLL/COMPASS subunit WDR5 (Figure 3). Knockdown of WDR5 resulted in a significant loss in both basal and inducible H3K4me3 at the MHC-II proximal promoter (Figure 3b), at CIITA pIV (Figure 3c) and at the GAPDH promoter (see Additional file 3).

### WDR5 does not contribute to histone H3K18 acetylation

**Figure 4:**
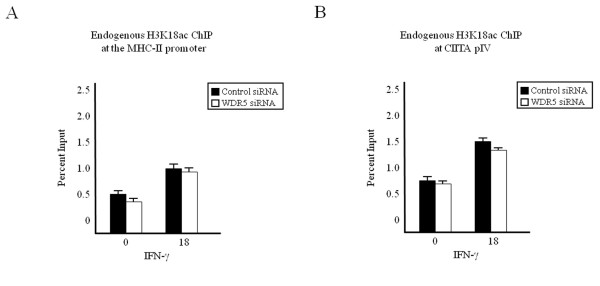
**WDR5 knockdown does not affect H3K18ac**. (a, b) HeLa cells transfected with scrambled control or WDR5-specific siRNA were stimulated with IFN-γ and subjected to ChIP assay. Lysates were immunoprecipitated with control or endogenous H3K18ac antibody. Associated DNA was isolated and analyzed via real-time PCR as described in Figure 2, using primers and probes specific for (a) MHC-II promoter or (b) CIITA pIV. Data are presented as percentage input. IgG Isotype control values were 0.06 ± 0.03 (MHC-II promoter) and 0.003 ± 0.002 (CIITA pIV). Values represent mean ± SEM of (n = 3) independent experiments.

The ATPases of the 19S regulator, which serves as the cap of the 26S proteasome, have been implicated in mediating a subset (H3K18ac, H3R17me2 and H3K4me3) of histone-activating modifications, independent of the canonical protein degradation role of the proteasome [36,41]. We have previously observed that WDR5 associates with histone-modifying enzymes known to mediate this subset of activating modifications including cAMP response element binding protein (CBP), coactivator-associated arginine methyltransferase (CARM)1 [36,41] and the HMTase SET1 [10,35]. To characterize the role of WDR5 in mediating additional histone modifications, we performed ChIP in cells transfected with WDR5 siRNA (Figure 4). Because CBP is capable of mediating H3K18ac [42,43], we determined by ChIP assay the levels of H3K18ac at the MHC-II proximal promoter (Figure 4a) and CIITA pIV (Figure 4b) in WDR5 knockdown cells. Levels of H3K18 acetylation in WDR5 knockdown cells (Figure 4a-b, white bars) were comparable to those in control siRNA-transfected cells (Figure 4a-b, black bars). WDR5 knockdown significantly decreased H3K4me3 but did not affect H3K18ac at the GAPDH promoter (see Additional file 3). As previously shown [36,41], neither cytokine stimulation nor siRNA transfection results in a loss of endogenous histone H3 (see Additional file 4). Therefore, although we had previously observed interaction of WDR5 with the HAT CBP, which is responsible for H3K18ac, and although both WDR5 and CBP binding are negatively affected by knockdown of the 19S ATPase Sug1, knockdown of WDR5 did not have a similar effect to that of Sug1 knockdown on CBP, as there was no effect on histone H3K18 acetylation. Thus, we looked further up the activation sequence to determine the role played by Sug1 in activating cytokine-inducible genes.

### H3K27me3 is enhanced upon Sug1 knockdown

**Figure 5:**
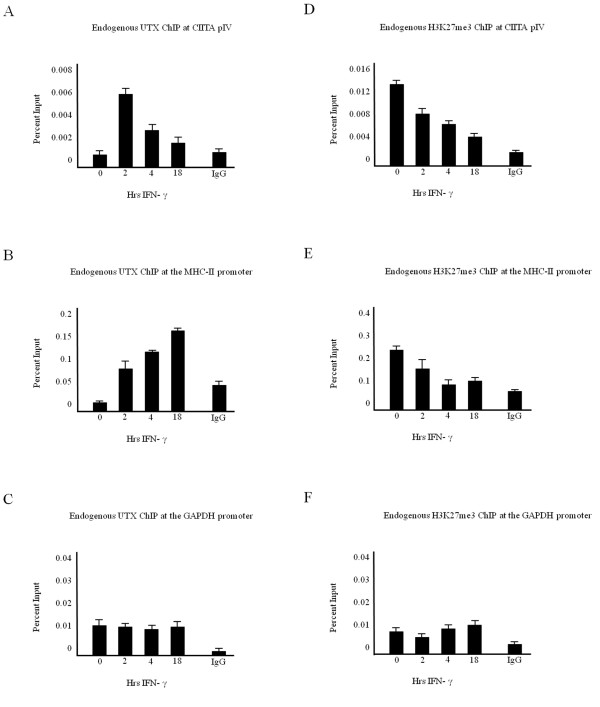
**UTX recruitment correlates with a reduction in H3K27 trimethylation**. (a-c) UTX associates with cytokine-inducible promoters. ChIP assays were carried out in HeLa cells stimulated with IFN-γ for 0 to 18 hours. Lysates were immunoprecipitated with control or endogenous UTX antibody. Associated DNA was isolated and analyzed via real-time PCR, using primers and probe spanning (a) CIITA pIV, (b) the MHC-II promoter or (c) the GAPDH promoter. Data are presented as percentage input. Values represent mean ± SEM of (n = 3) independent experiments. (d-f) H3K27 trimethylation was lost upon cytokine stimulation. ChIP assays were carried out in HeLa cells stimulated with IFN-γ for 0 to 18 hours. Lysates were immunoprecipitated with control or endogenous H3K27me3 antibody. Associated DNA was isolated and analyzed via real-time PCR using primers and probe spanning (d) CIITA pIV, (e) the MHC-II promoter or (f) the GAPDH promoter. Data are presented as percentage input. IgG isotype control values were 0.001 ± 0.0002 (CIITA pIV), 0.08 ± 0.02 (MHC-II promoter) and 0.001 ± 0.0009 (GAPDH promoter). Values represent mean ± SEM of (n = 3) independent experiments.

Evidence suggests that demethylation of silencing modifications is intimately linked to the deposition of histone-activating events to promote the relaxation of the repressive nature of chromatin. The recently identified H3K27me3 histone demethylase human UTX (ubiquitously transcribed tetratricopeptide repeat gene on X chromosome), a member of the Jumonji C family of proteins, is a di- and trimethyl H3K27 demethylase, which has been found in complex with common MLL/COMPASS subunits and associated with the elongating form of RNA polymerase II [14,15,44]. To determine if UTX associates with cytokine-inducible promoters, ChIP assays were performed for endogenous UTX recruitment to the inducible CIITA pIV (Figure 5a) and the MHC-II proximal promoter (Figure 5b). Low levels of UTX are bound to CIITA pIV in unstimulated cells (Figure 5a), which corresponds to elevated H3K27me3 in unstimulated cells (Figure 5d). Rapid recruitment of UTX to CIITA pIV is observed upon cytokine stimulation (Figure 5a), which correlates with reports of early chromatin remodeling events and rapid transcription factor recruitment to CIITA pIV [28,37,45]. Additionally, recruitment of UTX to the MHC-II promoter (Figure 5b) correlates with a reduction in H3K27me3 levels (Figure 5e). By contrast, UTX association remains relatively constant over a time course of IFN-γ at the constitutively active GAPDH promoter (Figure 5c), where levels of H3K27me3 are low (Figure 5f).

**Figure 6:**
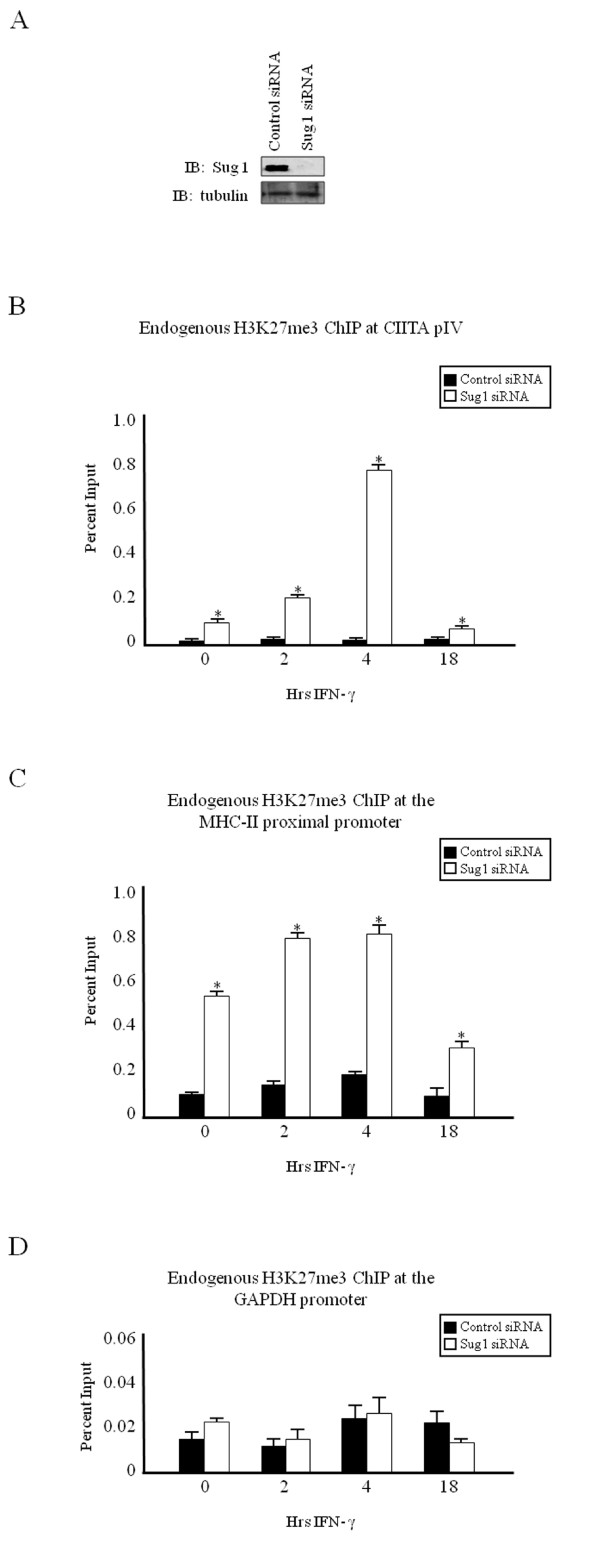
**H3K27me3 is enhanced upon Sug1 knockdown**. (a) Sug1 siRNA efficiently decreases endogenous Sug1. HeLa cells were transfected with control or Sug1-specific siRNA, harvested and subjected to western blot analysis for (top) endogenous Sug1 and (bottom) endogenous tubulin. (b-d) H3K27me3 is elevated at cytokine-inducible genes upon diminished Sug1 expression. ChIP assays were carried out in HeLa cells stimulated with IFN-γ for 0 to 18 hours. Lysates were immunoprecipitated with control or endogenous H3K27me3 antibody. Associated DNA was isolated and analyzed as in Figure 2 using primers and probe spanning (b) CIITA pIV, (c) the MHC-II proximal promoter and (d) the GAPDH promoter. Data are presented as percentage input. IgG isotype control values were 0.003 ± 0.001 (CIITA pIV), 0.08 ± 0.02 (MHC-II promoter) and 0.01 ± 0.005 (GAPDH promoter). Values represent mean ± SEM of (n = 3) independent experiments.

We previously observed that Sug1 regulates H3K18ac, H3R17me2 and H3K4me3 modifications independently from other localized enzymes [36]. These data suggested that the 19S proteasome may play a role in regulating multienzyme recruitment to cytokine-inducible promoters, but does not eliminate the possibility that 19S ATPases may regulate an additional upstream event that is a prerequisite for these events. Therefore, we sought to determine the degree of H3K27me3 in Sug1 knockdown cells. Chromatin immunoprecipitations in HeLa cells transfected with Sug1 siRNA (Figure 6b-c, white bars) show that H3K27me3 levels are substantially elevated over similarly treated control siRNA transfected cells (Figure 6b-c, black bars). At the non-bivalent GAPDH promoter, the lack of Sug1 fails to significantly affect H3K27me3 (Figure 6d). Together these data strongly suggest that the lack of Sug1 promotes a closed chromatin structure.

## Discussion

We previously demonstrated that the 19S proteasome regulates activating H3K4 trimethylation at cytokine-inducible promoters, potentially through interactions with common MLL/COMPASS subunits, as a loss of recruitment of WDR5 and Ash2L subunits is observed upon Sug1 knockdown [36]. In the current study, we showed that these common MLL/COMPASS subunits including WDR5, Ash2L and RbBP5 bind to both the MHC-II promoter and to CIITA pIV. The enrichment of recruited subunits upon IFN-γ stimulation varies, and the subunits bind with intensities similar to constitutively expressed GAPDH. Whether the increases in recruitment of these common subunits reflects increases in binding or differences in antibody affinity remains to be determined. One plausible explanation is that a subcomplex is assembled on promoters before transcription initiation. Overexpressing a single common MLL/COMPASS subunit significantly enhances promoter activity and gene expression, further implicating these subunits as co-activators for MHC-II. Consistent with this, we observed a loss of H3K4me3 at the MHC-II promoter and CIITA pIV when levels of endogenous WDR5 were diminished. However, as WDR5 has been found in complexes that lack SET domain histone methyltransferase enzymes, we cannot rule out the possibility that the loss of H3K4me3 observed upon WDR5 knockdown is due to other mechanisms [46].

We previously observed a loss in inducible WDR5 and Ash2L binding to CIITA pIV when endogenous Sug1 is diminished [36]. These findings implied that the 19S proteasome plays a role in stabilizing a large multienzyme histone remodeling complex through an interaction dependent on WDR5 at cytokine-inducible genes. Therefore, we sought to determine if knockdown of WDR5 mirrors that of 19S ATPase knockdown, which results in a loss of H3K18ac in addition to H3K4me3 [36,41,45]. Knockdown of WDR5 did not mirror that of Sug1 knockdown as there was no effect on histone H3K18 acetylation. Thus, the initial chromatin reorganization event regulated by 19S ATPases remains to be determined.

Evidence now suggests that demethylation of silencing modifications is intimately linked to the deposition of histone-activating events to promote the relaxation of the repressive nature of chromatin [14,15,47,48]. In addition to binding CBP and CARM-1, the common MLL/COMPASS subunit WDR5 is associated with histone H3K27me3 demethylases [14,15]. Consistent with this, we observed a gain of one of the known H3K27me3 demethylases (UTX) and a loss of H3K27me3 at CIITA pIV, and at the MHC-II promoter, upon cytokine induction [14]. As we previously observed that WDR5 and Ash2L binding to CIITA pIV is inhibited in the absence of Sug1, it was expected that histone demethylase recruitment would be negatively affected upon diminished Sug1 expression and would in turn affect regulation of H3K27me3. In fact, H3K27me3 levels are significantly elevated at each of these cytokine-inducible, bivalent promoters in Sug1 siRNA-transfected cells, but are unaffected at the constitutively active GAPDH promoter. As little is known regarding how inducible genes are relieved from and subsequently returned to a repressive state, the dynamic recruitment and activity of UTX and other histone H3K27me3 demethylases to cytokine-inducible promoters is not well understood. Although we show here that the 19S ATPase Sug1 functions to release chromatin from a repressive state, further studies are required to characterize the molecular interactions between 19S ATPases, UTX, JMJD3 and silenced chromatin.

To our knowledge, ours is the first report implicating the 19S proteasomal ATPases in regulating the initial chromatin remodeling events at cytokine-inducible promoters that releases surrounding chromatin from a more condensed, repressive state. Our observations that in the absence of Sug1, methylation of H3K27 is not only elevated, but is also persistent, strongly implicate the 19S ATPases in initial chromatin reorganization in response to stimuli. A full understanding of the contributions of Sug1 and the other 19S ATPases to the initial epigenetic regulation and chromatin reorganization at cytokine-inducible genes requires further studies into the molecular interactions occurring at these promoters.

## Methods

### Cell Lines

HeLa (human epithelial) cells (American Type Culture Collection, Manassas, VA, USA) were maintained in Dulbecco's modified Eagle's medium (DMEM; Mediatech Inc., Herndon, VA, USA) supplemented with 10% fetal calf serum, 5 mM L-glutamine and 5 mM penicillin-streptomycin at 37°C with 5% carbon dioxide.

### Antibodies

Trimethylated H3K4, acetylated H3K18 and WDR5 antibodies were from Abcam (Cambridge, MA, USA). Ash2L and RbBP5 antibodies were from Bethyl Laboratories (Montgomery, TX, USA). Rabbit IgG isotype control and mouse IgG isotype control antibodies were from Upstate (Lake Placid, NY, USA). Sug1 antibody was from Novus Biologicals (Littleton, CO, USA). Horseradish peroxidase (HRP)-conjugated mouse antibody (W4021) was from Promega (Madison, WI, USA) and HRP-conjugated rabbit antibody (1858415) was from Pierce (Rockland, IL, USA).

### Plasmids

Flag-CIITA, *HLA-DRA*-Luc, pcDNA3 plasmids were as previously described [49]. *Renilla *luciferase control vector (E2231) was from Promega (Madison, WI, USA). Myc-Sug1 was generously provided by A. A. Wani [50]. Flag-WDR5, Flag-Ash2L and Flag-RbBP5 were graciously provided by A. Shilatifard [35].

### siRNA constructs and transient transfections

Previously described siRNAs [41,49] were used for transient knockdown of the Sug1 19S ATPase. Pooled siRNA duplexes for WDR5 were purchased from Santa Cruz Biotechnology (Santa Cruz, CA, USA). HeLa cells transfected with Allstar scrambled sequence control siRNA (Qiagen, Valencia, CA, USA) or specific siRNA were treated with IFN-γ as indicated. Cells were lysed in NP40 lysis buffer (1 M Tris pH 8.0, 1 M KCl, 10% NP40, 0.5 M EDTA, 5 M NaCl, 1 M dithiothreitol, distilled (d)H_2_O) supplemented with Complete EDTA-free Protease Inhibitors (Roche, Indianapolis, IN, USA), and knockdown efficiency was assessed by immunoblotting as described above.

### Luciferase reporter assays

HeLa cells plated in six-well plates at a density of 5 × 10^4 ^cells/well were transfected with CIITA, *HLA-DRA*-Luc, *Renilla*, pcDNA3 and MLL/COMPASS subunit plasmids as indicated, using Fugene 6 (Roche) according to the manufacturer's instructions. Twenty-four hours after transfection, cells were subjected to dual-luciferase assay (Promega) according to the manufacturer's protocol.

### RNA transcript levels

HeLa cells were plated at a density of 8 × 10^5 ^cells/plate and transfected with 1 μg CIITA alone or in combination with MLL/COMPASS subunit plasmids (0.5 μg and 1 μg) as indicated, using Fugene 6 (Roche) according to the manufacturer's instructions. Twenty-four hours after transfection, cells were harvested and total RNA was prepared using TRIzol reagent (Invitrogen, Carlsbad, CA, USA) according to the manufacturer's instructions. Omniscript reverse transcription kit (Qiagen) was used to generate cDNA using gene-specific antisense primers for MHC-II *HLA-DR *[49] and GAPDH [51]. cDNA was quantified via real-time PCR using previously published primers and probes specific for MHC-II *HLA-DR *[49] and GAPDH [51]. Real-time PCR values were generated on the basis of standard curves generated for each gene and are presented as fold changes over non-transfected samples.

### ChIP assays

ChIP assays were performed as previously described [52]. In brief, HeLa cells were stimulated with 500 U/ml IFN-γ (Peprotech, Rocky Hill, NJ, USA) as indicated. Crosslinked cells were lysed in SDS lysis buffer (1% SDS, 10 mM EDTA, 50 mM Tris pH 8.0, dH_2_0) with Complete EDTA-free Protease Inhibitors (Roche) for 20 minutes on ice, and sonicated to generate sheared DNA with an average length of 500-750 bp. Sonicated samples were precleared with salmon sperm-coated agarose beads (Upstate), and half of the lysate was immunoprecipitated with 5 μg of polyclonal antibody against H3K4me3, H3K18ac, WDR5 (all Abcam), Ash2L or RbBP5 (both Bethyl Laboratories) overnight at 4°C. The remaining lysate was immunoprecipitated with isotype control antibody (Upstate). After a 2 hour incubation with 60 μl of salmon sperm-coated agarose beads, samples were washed for 5 minutes at 4°C with the following buffers: low salt buffer (0.1% SDS, 1% Triton X-100, 2 mM EDTA, 20 mM Tris pH 8.0, 150 mM NaCl, dH20), high salt buffer (0.1% SDS, 1% Triton X-100, 2 mM EDTA, 20 mM Tris pH 8.0, 500 mM NaCl, dH20), LiCl buffer (0.25 M LiCl, 1% NP40, 1% DOC, 1 mM EDTA, 10 mM Tris pH 8.0, dH20) and 1 × TE buffer, and were eluted with SDS elution buffer (1% SDS, 0.1 M NaHCO3, dH_2_O). After elution, crosslinks were reversed with 5 M NaCl at 65°C and immunoprecipitated, and control DNA was isolated using a phenol:chloroform:isoamyl alcohol mix (Invitrogen) according to the manufacturer's protocol. Isolated DNA was analyzed by real-time PCR using probes and primers spanning the W-X-Y box of the MHC-II *HLA-DRA *promoter and CIITA pIV [36,41,49]. Values graphed were calculated based on standard curves generated.

#### ChIP in siRNA knockdown cells

HeLa cells were transfected with specific siRNA or control siRNA using RNAiFect transfection reagent (all from Qiagen) according to the manufacturer's instructions. Cells were treated with 500 U/ml IFN-γ as indicated. 10% of the total cell volume was lysed in 1% Nonidet P-40 buffer (1 M Tris pH 8.0, 1 M KCl, 10% NP40, 0.5 M EDTA, 5 M NaCl, 1 M DTT, dH_2_O) with Complete EDTA-free Protease Inhibitors (Roche) and analyzed by immunoblotting for knockdown verification. The remaining fraction of cells was subjected to ChIP assay.

## Competing interests

The authors declare that they have no competing interests.

## Authors' contributions

OIK carried out the ChIP assays, participated in the reporter assays, participated in coordination of the study and helped to draft the manuscript. NTM, ADT, RKD and JKB carried out the reporter and gene expression assays and participated in the ChIP assays. SFG conceived of the study, participated in its design and coordination, and helped to draft the manuscript. All authors read and approved the final manuscript.

## Supplementary Material

Additional file 1 **Supplemental Figure 1. **IFN-γ inducible CIITA promoter IV (pIV) transcription drives expression of MHC-II. Before IFN-γ stimulation, both MHC-II and CIITA pIV exhibit low to moderate acetylation of histone H3 and H4 and are occupied by ubiquitiously expressed factors. MHC-II is bound by an enhanceosome complex of nuclear factor Y (NFY), regulatory factor X (RFX) and CREB, and pIV is bound in a highly conserved E-box by upstream stimulating factor (USF)-1 and c-Myc. Upon stimulation with the pro-inflammatory cytokine IFN-γ, the JAK/STAT1 pathway is triggered, leading to enhanced pIV acetylation and methylation, to rapid recruitment of the STAT1 homodimer to the pIV GAS element and to IFN response factors 1 and 2 (IRF1/2) binding to the pIV IRE. Once expressed, CIITA binds each component of the enhanceosome complex and recruits basal transcriptional components to initiate the switch to an elongation complex. Click here for file

Additional file 2 **Supplemental Figure 2. **Common MLL/COMPASS subunits associate with the GAPDH promoter. **(a-c) **ChIP assays were carried out in HeLa cells stimulated with IFN-γ for 0 to 18 hours. Lysates were immunoprecipitated with control or endogenous **(a) **WDR5, **(b) **Ash2L or **(c) **RbBP5 antibody. Associated DNA was isolated and analyzed via real-time PCR using primers and probe spanning the GAPDH promoter. Data are presented as percentage input. Values represent mean ± SEM of (n = 3) independent experiments. IgG isotype control values were 0.001 ± 0.0005 Click here for file

Additional file 3 **Supplemental Figure 3.** Knockdown of a common MLL/COMPASS subunit decreases H3K4me3 but not H3K18ac at the GAPDH promoter. (a, b) HeLa cells transfected with scrambled control or WDR5-specific siRNA were stimulated with IFN-γ and subjected to ChIP assay. Lysates were immunoprecipitated with control, (a) endogenous H3K4me3 or (b) endogenous H3K18ac antibody. Associated DNA was isolated and analyzed via real-time PCR as described in Figure 1, using primers and probes specific for the GAPDH promoter. Data are presented as percentage input. IgG Isotype control values were 0.1 ± 0.05. Values represent mean ± SEM of (n = 2-3) independent experiments. Click here for file

Additional file 4 **Supplemental Figure 4.** Neither IFN-γ stimulation nor siRNA transfection affect levels of histone H3. **(a, b) **HeLa cells stimulated with (a) IFN-γ or (b) transfected with scrambled control or WDR5-specific siRNA were subjected to ChIP assay. Lysates were immunoprecipitated with control or endogenous H3 antibody. Associated DNA was isolated and analyzed via real-time PCR as described in Figure 2 using primers and probes specific for CIITA pIV. Data are presented as percentage input. Values represent mean ± SEM of (n = 2-3) independent experiments. Click here for file
