# Supplementary figures and images for: Roles for common MLL/COMPASS subunits and the 19S proteasome in regulating CIITA pIV and MHC class II gene expression and promoter methylation
Source: Epigenetics Chromatin. 2010 Feb 4;3:5. doi: 10.1186/1756-8935-3-5 (PMC2829561; doi:10.1186/1756-8935-3-5)

Supplemental Figure 1

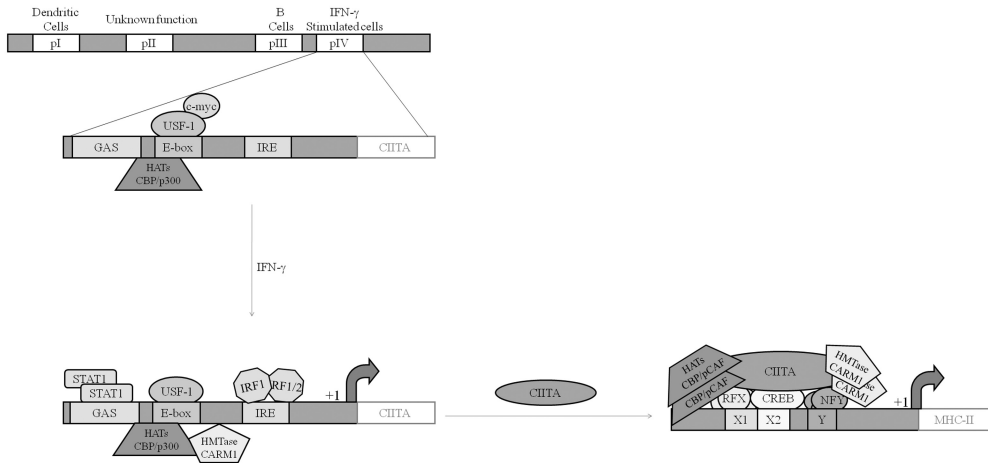

Supplement: Additional file 1 — Supplemental Figure 1. IFN-γ inducible CIITA promoter IV (pIV) transcription drives expression of MHC-II. Before IFN-γ stimulation, both MHC-II and CIITA pIV exhibit low to moderate acetylation of histone H3 and H4 and are occupied by ubiquitiously expressed factors. MHC-II is bound by an enhanceosome complex of nuclear factor Y (NFY), regulatory factor X (RFX) and CREB, and pIV is bound in a highly conserved E-box by upstream stimulating factor (USF)-1 and c-Myc. Upon stimulation with the pro-inflammatory cytokine IFN-γ, the JAK/STAT1 pathway is triggered, leading to enhanced pIV acetylation and methylation, to rapid recruitment of the STAT1 homodimer to the pIV GAS element and to IFN response factors 1 and 2 (IRF1/2) binding to the pIV IRE. Once expressed, CIITA binds each component of the enhanceosome complex and recruits basal transcriptional components to initiate the switch to an elongation complex. [file 1756-8935-3-5-S1.PDF]

# Supplemental Figure 3

A

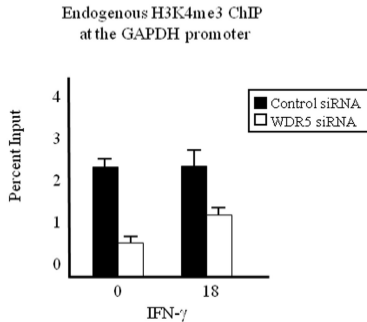

B

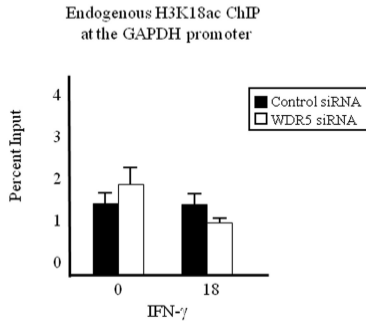

Supplement: Additional file 3 — Supplemental Figure 3. Knockdown of a common MLL/COMPASS subunit decreases H3K4me3 but not H3K18ac at the GAPDH promoter. (a, b) HeLa cells transfected with scrambled control or WDR5-specific siRNA were stimulated with IFN-γ and subjected to ChIP assay. Lysates were immunoprecipitated with control, (a) endogenous H3K4me3 or (b) endogenous H3K18ac antibody. Associated DNA was isolated and analyzed via real-time PCR as described in Figure 1, using primers and probes specific for the GAPDH promoter. Data are presented as percentage input. IgG Isotype control values were 0.1 ± 0.05. Values represent mean ± SEM of (n = 2-3) independent experiments. [file 1756-8935-3-5-S3.PDF]
