# Supplementary material for: Roles for common MLL/COMPASS subunits and the 19S proteasome in regulating CIITA pIV and MHC class II gene expression and promoter methylation
Source: Epigenetics Chromatin. 2010 Feb 4;3:5. doi: 10.1186/1756-8935-3-5 (PMC2829561; doi:10.1186/1756-8935-3-5)

Supplemental Figure 2

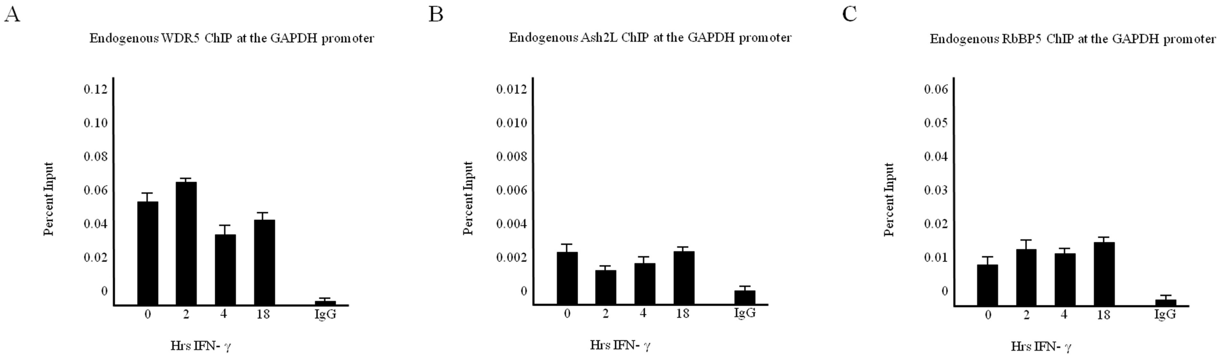

Supplement: Additional file 2 — Supplemental Figure 2. Common MLL/COMPASS subunits associate with the GAPDH promoter. (a-c) ChIP assays were carried out in HeLa cells stimulated with IFN-γ for 0 to 18 hours. Lysates were immunoprecipitated with control or endogenous (a) WDR5, (b) Ash2L or (c) RbBP5 antibody. Associated DNA was isolated and analyzed via real-time PCR using primers and probe spanning the GAPDH promoter. Data are presented as percentage input. Values represent mean ± SEM of (n = 3) independent experiments. IgG isotype control values were 0.001 ± 0.0005 [file 1756-8935-3-5-S2.PDF]
