# Supplementary material for: Roles for common MLL/COMPASS subunits and the 19S proteasome in regulating CIITA pIV and MHC class II gene expression and promoter methylation
Source: Epigenetics Chromatin. 2010 Feb 4;3:5. doi: 10.1186/1756-8935-3-5 (PMC2829561; doi:10.1186/1756-8935-3-5)

Supplemental Figure 4

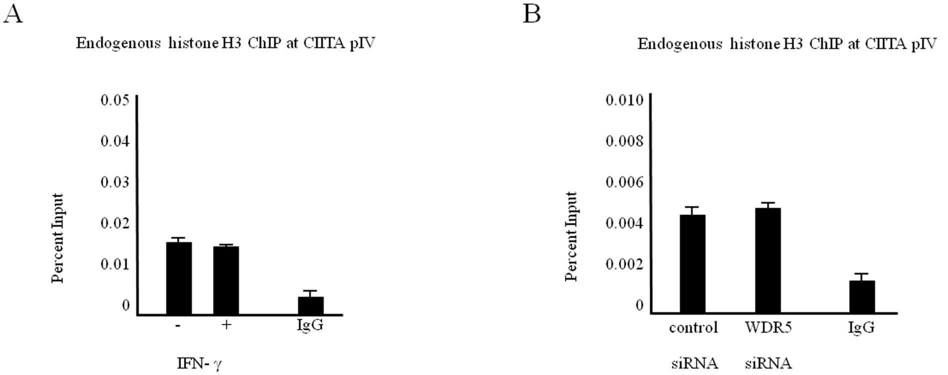

Supplement: Additional file 4 — Supplemental Figure 4. Neither IFN-γ stimulation nor siRNA transfection affect levels of histone H3. (a, b) HeLa cells stimulated with (a) IFN-γ or (b) transfected with scrambled control or WDR5-specific siRNA were subjected to ChIP assay. Lysates were immunoprecipitated with control or endogenous H3 antibody. Associated DNA was isolated and analyzed via real-time PCR as described in Figure 2 using primers and probes specific for CIITA pIV. Data are presented as percentage input. Values represent mean ± SEM of (n = 2-3) independent experiments. [file 1756-8935-3-5-S4.PDF]
